# Generation of human induced pluripotent stem cell-derived cardiomyocytes in 2D monolayer and scalable 3D suspension bioreactor cultures with reduced batch-to-batch variations

**DOI:** 10.7150/thno.32058

**Published:** 2019-09-25

**Authors:** Sarkawt Hamad, Daniel Derichsweiler, Symeon Papadopoulos, Filomain Nguemo, Tomo Šarić, Agapios Sachinidis, Konrad Brockmeier, Jürgen Hescheler, Bastiaan J Boukens, Kurt Pfannkuche

**Affiliations:** 1 Center for Physiology and Pathophysiology, Institute for Neurophysiology, University of Cologne, Medical Faculty, Cologne, Germany; 2 Biology Department, Faculty of Science, Soran University, Soran, Kurdistan region-Iraq; 3 Center for Physiology and Pathophysiology, Institute for Vegetative Physiology, University of Cologne, Medical Faculty, Cologne, Germany; 4 Department of Medical Biology, Amsterdam University Medical Center, Amsterdam, The Netherlands; 5 Department of Pediatric Cardiology, University Clinics of Cologne, Cologne, Germany

**Keywords:** Human induced pluripotent stem cells, iPS cells, hiPSCs, differentiation, cardiomyocytes, regenerative medicine, bioreactor suspension culture, Wnt signaling, ascorbate, robust method

## Abstract

Human induced pluripotent stem cell derived cardiomyocytes (hiPSC-CMs) are promising candidates to treat myocardial infarction and other cardiac diseases. Such treatments require pure cardiomyocytes (CMs) in large quantities.

**Methods**: In the present study we describe an improved protocol for production of hiPSC-CMs in which hiPSCs are first converted into mesodermal cells by stimulation of wingless (Wnt) signaling using CHIR99021, which are then further differentiated into CM progenitors by simultaneous inhibition of porcupine and tankyrase pathways using IWP2 and XAV939 under continuous supplementation of ascorbate during the entire differentiation procedure.

**Results**: The protocol resulted in reproducible generation of >90% cardiac troponin T (TNNT2)-positive cells containing highly organized sarcomeres. In 2D monolayer cultures CM yields amounted to 0.5 million cells per cm^2^ growth area, and on average 72 million cells per 100 mL bioreactor suspension culture without continuous perfusion. The differentiation efficiency was hardly affected by the initial seeding density of undifferentiated hiPSCs. Furthermore, batch-to-batch variations were reduced by combinatorial use of ascorbate, IWP2, and XAV939.

**Conclusion**: Combined inhibition of porcupine and tankyrase sub-pathways of Wnt signaling and continuous ascorbate supplementation, enable robust and efficient production of hiPSC-CMs.

## Introduction

The pumping function of the heart is of vital importance to generate sufficient blood flow and pressure. Different pathologies impair cardiac function; cardiac infarction results in an acute loss of large quantities of contractile cells and fibrotic remodeling of the tissue. As a result heart failure can occur. Adult cardiomyocytes (CMs) do not divide and therefore novel approaches need to be identified to overcome the minor regenerative capacity of the heart, provide new CMs to the diseased organ and reduce the size of the scar and hence the risk of heart failure. Today, neither pharmacological nor advanced surgical interventions are capable of restoring the lost contractile function of the infarcted area 1. Human induced pluripotent stem cell (hiPSC)-based technologies are promising candidates to reach a suitable treatment for partially recovering damaged cardiac tissue 2.

Human iPSCs have been discovered in 2007 3. They are highly similar to embryonic stem cells, differentiate into a variety of different cell types *in vitro* and generate teratomas* in vivo* 3. Several groups demonstrated that CMs derived from pluripotent stem cells (PSCs) can be used for treating heart diseases, including myocardial infarction 4-9. Currently, these attempts still remain in the stage of pre-clinical investigations, due to several open questions, including high incidence of arrhythmia after transplantation of induced pluripotent stem cell derived cardiomyocytes (iPSC-CMs), restricted access to efficient differentiation protocols and lack of long-term studies in large animals to provide sufficient data on the safety of the method in the long term 10.

*In vivo* physiological pathways are adapted for differentiating CMs at early stage *in vitro* and biological morphogens are replaced by synthetic small molecules that can be produced at comparable low costs 11. Therefore, directed hiPSCs differentiation into CMs has gained increasing attention in cardiac regenerative medicine 12, 13. Initially, bone morphogenetic protein 4 and activin A were utilized for production of hiPSC-CMs, but the differentiation efficiency hardly exceeded 30% 14. A sandwich culture of pluripotent stem cells between layers of Matrigel resulted in very high efficiencies of about 98% but was not broadly applied because of low scalability of this method 15. Dickkopf-related protein 1, or small molecules including IWP4, IWP2, IWR1, and XAV939 have been used for manipulating wingless (Wnt) signaling and replacing BMP4 in the differentiation process 16, 17. Application of those small molecules improved the CM purity, and increased the total CM yield 11, 18. However, there are still obstacles to be solved, including fluctuations in differentiation efficiency resulting from variability in initial cell seeding at the initiation of differentiation, insufficient CM purity in scalable approaches, and difficulties in the upscaling process itself 19, 20. Therefore, this investigation focuses on the development of a robust protocol for differentiating hiPSCs into CMs, in both, monolayer and controlled suspension culture conditions that is not affected by the variability in cell seeding density. We hypothesize that combined blocking of porcupine and tankyrase pathways of Wnt signaling reduces experimental variability and increases purity of hiPSC-CMs. Our results show that the combined inhibition of the aforementioned pathways by IWP2 and XAV939 in combination with continuous supplementation of ascorbate results in a hiPSC-CM purity of >90% with minor inter-experimental variations and low sensitivity to the initial hiPSC seeding density.

## Materials and Methods

### Human pluripotent stem cell culture

Human iPSC lines used in this study were the NP0040-8 (male) and NP0141-31B (female) cell lines that were generated by Saric group, and the IMR90 cell line that was kindly provided by WiCell (https://www.wicell.org/). Detailed information about the NP0040-8 and NP0141-31B cell lines can be obtained from the online hPSC line registry hPSCreg (https://hpscreg.eu/) under names UKKi011-A and UKKi032-C, respectively. hiPSC lines were cultured in E8 medium composed of DMEM / F12 (1:1) + Glutamax (Thermo Fisher Scientific, # 31331-028) supplemented with 64 μg/mL L-ascorbic acid phosphate magnesium n-hydrate (Wako Chemicals Europe, # 013-12061), 20 μg/mL insulin (Lilly Deutschland GmbH, “Humalog 100I.E.”), 5 μg/mL transferrin (Sigma-Aldrich, # T3705), 14 ng/mL sodium selenite (Sigma-Aldrich, # S5261), 100 ng/mL heparin sodium salt (Sigma-Aldrich, # H3149), 100 ng/mL fibroblast growth factor 2 (Peprotech, # 100-18B), and 2 ng/mL transforming growth factor β (Peprotech, # 100-21) 21 on six-well plates coated with 10 μg/cm^2^ Matrigel Matrix (hESC-qualified, Corning, # 734-1440) for one hour at room temperature, and incubated in a humidified incubator at 37°C, with 5% CO_2_. Cells were passaged every 4 days at 1:20 splitting ratio using 0.5 mM EDTA (Sigma-Aldrich, # 6381-92-6). Following dissociation of hiPSCs E8 medium was supplemented by 5 μM of Rho Kinase (ROCK) inhibitor (Y27632, Adooq, # A11001-5) for the first two days after passage. Medium was changed every other day, and 0.5 mL penicillin and streptomycin (Gibco, # 15140-122) per mL culture medium were supplemented in both hiPSCs and hiPSC-CM culture media.

### Generation of hiPSC-CM in 2D monolayer culture

A state-of-the-art differentiation protocol for hiPSC-CMs using small molecules to induce differentiation was adapted from Lian and colleagues 22. Briefly, for optimizing seeding density of hiPSCs 0.5, 0.75, 1.0, 1.25, or 1.5 million cells were plated per well of Matrigel coated 12 well plates in 2 mL E8 medium/well for four days. On the first day ROCK inhibitor was applied, the three following days E8 medium without ROCK was used as growth medium. Use of E8 is not in line with the above cited protocol that describes hiPSC culture in mTESR1 medium. We used E8 for initial expansion of the hiPSCs because our cell lines are adapted to this medium for expansion. Cells were incubated in a humidified incubator at 37°C, with 5% CO_2_. Once culture confluency had reached 80% the medium was changed into RPMI 1640 (Thermo Fisher Scientific, # 61870-010) with 1× B27 supplement without insulin (Thermo Fisher Science, # 175044) and 8 μM CHIR99021 (LC Laboratories, # C-6556) for 24 hours. (Lian and colleagues recommend titration of CHIR99021 in the range of 8 to 12µM. We used 8 µM here, because this concentration was proven to successfully induce differentiation in our optimized protocol.) After 24 hours, culture medium was changed into RPMI 1640 with 1× B27 supplement without insulin for 48 hours. 72 hours post CHIR99021 supplementation the culture medium was changed into RPMI 1640 with 1× B27 supplement without insulin and 5µM IWP_2_ (Tocris, # 3533) for 48 hours. On day 5, 7, and every three other days medium was changed in RPMI 1640 with 1× B27 supplement without (till day 7) and with insulin (from day 7 onwards), the cardiomyocyte contraction was observed on day 12.

Our optimized protocol started from washing hiPSCs culture with Dulbecco's phosphate buffered saline without calcium and magnesium (DPBS-/-), and 1 mL of 0.5 mM EDTA (room temperature) was added into each well of a six-well plate and incubated in a humidified incubator at 37°C, with 5% CO_2_. The incubation time for dissociation of hiPSCs was 3 minutes to obtain small clusters and 7 minutes for single cells. Either, 1:20 splitting ratio of small clusters, or 0.01, 0.02, 0.04, 0.06, 0.08, 0.1, 0.2, 0.3 and 0.4×10^6^ cells/cm^2^ were seeded on Matrigel-coated six-well plates and maintained in 2 mL of E8 medium for four days. For the first two days the culture medium was supplemented with 5 μM ROCK inhibitor. After four days the culture medium was changed to RPMI 1640 with 1× B27 supplement without insulin (Thermo Fisher Scientific, # A18956-01), 50 μg/mL ascorbate and 8 μM CHIR99021. This day was defined as day 0 of differentiation. Exactly after 24 hours, the medium was completely changed to RPMI 1640 with 1X B27 supplement without insulin, and 50 μg / mL ascorbate. At day 3, 72 hours after initiation of differentiation half of the medium was changed to RPMI 1640 with 1× B27 supplement without insulin, 50 μg / mL ascorbate, and final concentrations of 5 μM IWP2 (Tocris, # 3533 / 10), and 5 μM XAV939 (Sigma-Aldrich, # X3004) for 48 hours. From day 5 onwards the culture medium was changed to RPMI 1640 plus 1× B27 supplement without insulin and with 50 μg/mL ascorbate and replaced every 3 days.

### Generation of hiPSC-CM in 3D bioreactor suspension culture

**Bioreactor preparation:** Bioreactor (250 mL total volume) inner vessel walls were siliconized with 1 mL Sigmacote (Sigma-Aldrich, # SL2). Vessels were gently swirled to cover the whole surface with Sigmacote. The remaining Sigmacote was collected and reused. Coated vessels were left under the fume hood for one hour to remove the residual amount of liquid Sigmacote.

To prepare the bioreactor system (DASGIP Parallel Bioreactor Systems, Eppendorf) a calibration of the pH sensor was performed at pH 4 and 7. All bioreactor parts were assembled including: glass vessel, dissolved oxygen (DO) sensor, pH sensor, and head caps. The vessel was autoclaved at 121°C for 15 minutes, and cooled to room temperature. Under a laminar flow hood 100 mL of E8 medium were added to each of the 250 mL volume capacity glass vessel (Eppendorf; DASGIP Advanced Spinner Line DS, # DS0200TPSS). The vessel was placed back onto the temperature controller. Overnight polarization and calibration of DO sensor were performed at 60 revolutions per minute (rpm) agitation with 21% O_2_, 5% CO_2_ at 37°C and three standard liters per hour (sL / h) overlaying flow gas. After differentiation of CMs, the bioreactor vessels were washed with distilled water and 70% ethanol.

**hiPSC-CM differentiation:** hiPSCs were cultured on Matrigel-coated 100 mm dishes (Corning Life Science, # 353003) and incubated in a humidified incubator at 37°C, with 5% CO_2_. hiPSCs were cultured in E8 medium for about one week with daily medium change until 85% cell confluency was reached. Cells were washed with DPBS-/-. For dissociation, cells were incubated with 1-2 mL 0.5 mM EDTA per plate for 7 minutes at room temperature, the dissociated cells were passed through a 40 μm filter (Greiner Bio-One, # 542040) and collected in a 50 mL Falcon tube with 10 mL E8 medium containing 10 μM ROCK inhibitor. Cells were counted by an automatic cell counter (NaNoEnTek, Korea). 40 million single cells were re-suspended in 25 mL E8 medium supplemented with 10 μM of ROCK inhibitor and this suspension was inoculated into the bioreactor vessel containing 100 ml E8 medium containing 10 μM of ROCK inhibitor in order to obtain a final volume of 125 mL per vessel. Cultures were agitated at a propeller speed of 60 rpm, passed with 21% O_2_ and 5% CO_2_ by 3 sL/h overlay gassing and maintained at 37°C. After 48 hours of incubation (day -4 and -3) in E8 medium supplemented with ROCK inhibitor, the medium was changed to E8 medium without ROCK inhibitor and hiPSC clusters were incubated for additional 48 hours (day -2 and -1) until initiation of differentiation.

At day 0, the cardiac differentiation was induced by a complete change of the medium to RPMI 1640 supplemented with 1× B27 without insulin, 50 μg/mL ascorbate and 8 μM CHIR99021. After 24 hours, the complete medium was changed into RPMI 1640 containing 1× B27 without insulin and 50 μg/mL ascorbate, and cells were incubated for another 48 hours. At day 3, half of the medium was removed and replaced with fresh RPMI 1640 supplemented with 1× B27 without insulin, 100 μg/mL ascorbate, 10 μM IWP2 and 10 μM XAV939 for 48 hours. From day 7 of differentiation onwards, the medium was replaced every three days with fresh RPMI 1640 supplemented with 1× B27 without insulin and 50 μg/mL ascorbate. At day 18 of differentiation, CM clusters were collected in a 50 mL Falcon tube and allowed to settle on the bottom of a tube by gravity. Clusters were then washed with DPBS-/- and dissociated by using 0.05% Trypsin-EDTA (Thermo Fisher Scientific, # 25300-054). The dissociated cells were passed through an EASYstrainer filter with 40 μm pore size and single cells were counted by an automatic cell counter.

### Characterization of hiPSC-CMs by immunofluorescent staining

Dissociated single cells (1×10^6^) were plated on Matrigel-coated round cover slips in 12-well plates and incubated for two days in a humidified incubator at 37°C and CO_2_ level of 5% in 1 mL/well RPMI 1640 supplemented with 1× B27 without insulin and 50 μg/mL ascorbate. Alternatively, immunocytochemistry was performed directly with hiPSC-CMs that were generated on Matrigel-coated square cover slips in six well plates. The wells were washed with 1× DPBS-/-, fixed with 4% paraformaldehyde (PFA) for 15 minutes at room temperature, and permeabilized with 0.5% Triton X-100 in DPBS-/- for 15 minutes. Intact cardiac clusters were fixed and permeabilized with 100% methanol for 15 minutes. Unspecific antibody binding sites were blocked by incubation in 1 mL per well (or for clusters, in 1 mL per 15 ml tube) of 3% bovine serum albumin (BSA) (Sigma-Aldrich, # A2153) in DPBS-/- for one hour at room temperature. For preparation of cryoslices, cardiac clusters were embedded in Tissue-Tek (Sakura, # 1227900003) and stored frozen at -80°C, and cryo-sliced at 8 µm thickness. Cells or cardiac cluster samples were incubated overnight at 4°C in 3% BSA containing 1:200 dilution of troponin T (TNNT2) rabbit polyclonal IgG (Abcam, # ab45932) and 1:200 dilution of α-actinin (ACTN2) mouse monoclonal IgG1 (Sigma-Aldrich, # A7811). The next day, cells were washed three times with DBPS-/- and incubated for 60 minutes at room temperature in the dark and 3% BSA containing Hoechst 33342 and Alexa Fluor 555 goat α-rabbit IgG (Thermo Fisher Scientific, # A21430) and Alexa Fluor 647 goat α-mouse IgG (Thermo Fisher Scientific, # A21236), both at a concentration of 1 µg/ml. Following the final washing steps, cells were mounted with 5 µL SlowFade^TM^ Diamond Antifade Mountant (Thermo Fisher Scientific, # S36972) and examined by confocal fluorescent microscopy (SP8, Leica).

### Characterization of hiPSC-CMs by flow cytometry

1.5×10^6^ single cells were added into a 15 mL Falcon tube, washed with 1 mL DPBS-/-, and centrifuged at 120×*g* for 2 minutes. The supernatant was discarded, the cells fixed in 1% PFA for 15 minutes, and centrifuged at 120×*g* for 2 minutes. Cells were re-suspended in 1 mL of 90% methanol (-20°C) and left for 20 minutes at 4°C for further fixation and permeabilization. Cells were centrifuged at 120×*g* and washed by 2 mL DPBS-/- to remove the residue of methanol and then the pellet was incubated for one hour, or overnight in 100 µL of blocking buffer (0.5% BSA and 0.1% Triton X-100 in DPBS-/-) containing 1:50 dilution of troponin T (TNNT2) mouse monoclonal IgG2a (Santa Cruz Biotechnology, # Sc-20025) or normal mouse monoclonal IgG2a (Santa Cruz Biotechnology, # 3878), while for double staining Anti-Cardiac Troponin T-FITC (Miltyeni Biotec, # 130-119-575) and Anti-α-Actinin (Sarcomeric)-PE (Miltyeni Biotec, # 130-106-998), or Anti-Myosin regulatory light chain 2 atrial isoform conjugated with allophycocyanin (Anti-MLC2a-APC) (Miltyeni Biotec, # 130-118-674) and Anti-Myosin regulatory light chain 2 ventricular isoform conjugated with phycoerythrin (Anti-MLC2v-PE) (Miltyeni Biotec, # 130-119-680) were used. After washing in blocking buffer the cells were incubated for 30 minutes in 100 µL of blocking buffer containing 1 µg/mL secondary antibody Alexa Fluor 555 goat anti-mouse IgG (H+L) (Thermo Fisher Scientific, # A21422) in the dark and at room temperature. Cells were washed two to three times with 2 mL of DPBS-/-, the final cell pellet was re-suspended in 250 μL DPBS-/- and cells analyzed by flow cytometry (LSR Fortessa Analyzer, BD Biosciences). Data was evaluated using FCS express 6 (De Novo Software, Glendale, CA).

### Global gene expression analysis

Total RNA from hiPSC-CMs was isolated by PureLink RNA Mini Kit (Life technologies, # 12183018A). Briefly, 1 x 10^6^ hiPSC-CMs were added into RNase free 1.5 tubes, washed by PBS (-/-), and centrifuged at 300 g for 2 minutes at 4 °C. The pellet was re-suspended in 1 mL TRIzol (Life technologies, # 15596026), repetitive pipetting and mixing on a vortex mixer were performed to lyse CMs for 30 - 60 seconds. Lysate was incubated for 5 minutes at RT. For phase separation of RNA from phenol 0.2 mL chloroform (Sigma, # C-2432) per tube were added and carefully mixed by hand and centrifuged at 12000g for 15 minutes at 4 °C. 350 μL of colorless supernatant were transferred into new RNase free 1.5 mL tubes and 350 µL 100% ethanol (Carl Roth, # 9065.3) were added, and vortexed well. 700 μL of mixed sample were passed through a spin cartridge with collection tube (provided with the kit), and centrifuged at 12000 g for 1 minute at RT. The spin cartridge was washed by 500 µL wash buffer II (Life technologies, #12183018A) and centrifuged at 12000 g for 15 second at RT. 22 μL RNase free water were used to elute RNA into 1.5 RNase free tubes, RNA aliquots were stored at -80 °C.

For transcriptome analysis 5.5 µg fragmented biotin-labeled ds cDNA from cardiomyocytes were hybridized to Clariom™ S arrays (Clariom™ S arrays, human Applied Biosystems by Thermo Fisher Scientific). After staining, arrays were scanned with Affymetrix Gene-Chip Scanner-3000-7G while quality control analysis was performed using Affymetrix GCOS software. Transcriptome analysis was done at the transcriptomics core facility at the Center for Molecular Medicine Cologne (CMMC).

### Measurement and analysis of calcium transients

At day 18 of CM differentiation 1.2 X 10^6^ hiPSC-CMs were seeded on 10 µg / cm^2^ glass bottom dishes (MatTek corporation, # P35G-0-14-C) pre-coated with Matrigel and incubated until day 20 - 40 in a humidified incubator at 37°C and CO_2_ level of 5%. Per well 1 mL RPMI 1640 supplemented with 1× B27 without insulin and 50 μg/mL ascorbate was added, complete medium change was performed every other day. At day 20, 30, and 40 RPMI 1640 was discarded and hiPSC-CMs were incubated in HEPES-buffered Tyrode´s solution supplemented with sodium pyruvate. Cells were loaded for 30 minutes at 22 - 23 °C with 5 μM Fluo-4 AM calcium indicator (Thermo Fisher Scientific, #F14201), and measurements were conducted using an Olympus FluoView1000 confocal system. To examine the influence of adrenergic stimulation on frequency and kinetics of calcium transients, confocal scans were performed before and after addition of isoproterenol (Iso; Sigma, # I-5627) to a concentration of 10µM.

### Monitoring of hiPSC-CMs beating activity

hiPSC-CMs generated in 2D and 3D culture were dissociated at day 18 of differentiation. 0.15 X 10^6^ hiPSC-CMs were seeded on 10 µg / cm^2^ Matrigel pre-coated “E-plate Cardio 96” multiwell plates containing gold electrode arrays fused to the bottom of the plates (ACEA Biosciences) and incubated for further 6 - 7 days in 0.150 mL / well RPMI 1640 supplemented with 1× B27 without insulin and 50 μg/mL ascorbate. The E-plate was transferred into an xCELLigence Real Time Cell Analyser Cardio (RTCA) instrument (ACEA Biosciences, San Diego, USA) to monitor hiPSC-CMs contracting activity for 4 - 5 days. The measurement is based on fluctuations of the impedance of the cell layer when CMs contract in a syncytium. Once the syncytium monolayer hiPSC-CMs contractions were observed, the hiPSC-CMs were exposed to 10 µM Iso, and 10 µM carbachol (CCH) in two different row of E-plate for two days.The hiPSC-CMs beating rate was monitored for totally 9 days (2 days with drugs) and analyzed using the RTCA Cardio software version 1.0 (ACEA Biosciences).

### Electrophysiological characterization of monolayer

hiPSC-CMs were seeded on Matrigel-coated round cover slips (diameter of 2 cm) at a density of 1.7-2.3 x 10^6^ / cm^2^, and cultured inside 12-well plates in 2 mL RPMI 1640 supplemented with 1× B27 without insulin and 50 μg/mL ascorbate at 37 °C and 5% CO_2_. After 3 (n=3) or 11 days (n=4), the cover slips with hiPSC-CM were incubated for 15 min with a membrane potential-sensitive fluorescent dye (1uM DI-4-ANEPPS) and Blebbistatin (10 uM) to remove motion artefacts. After that the coverslips were placed in the optical mapping setup and superfused with Tyrode's solution (36±0.2˚C) containing (in mM): NaCl 140, KCl 5.4, CaCl2 1.8, MgCl2 1.0, glucose 5.5, and HEPES 5.0 (pH7.38). The monolayers were stimulated at 2 beat per minute at twice diastolic threshold with a pulse width of 2 ms using an extra cellular electrode. Optical action potentials were recorded using a CMOS camera (MICAM Ultima 100×100 pixels, SciMedia USA Ltd) and analyzed using custom-made software 23.

### Statistical analysis

GraphPad Prism software version 5 was used for statistical analysis and graph drawing. Where stated, graphs were created using the Olympus FluoView analysis software. De Novo software version FCS Express 6 was used for flow cytometry analysis. One way analysis of variance (ANOVA), and student unpaired t-test were used to test statistical difference between two groups. Bonferroni's test was chosen as a post hoc test. Data were represented as mean ± standard deviation (mean ± SD) when biological independent replications was three to six (n =3-6) and significant difference value was less than 0.05 (P < 0.05).

## Results

### Generation of hiPSC-CMs in 2D monolayer culture

**Figure 1:**
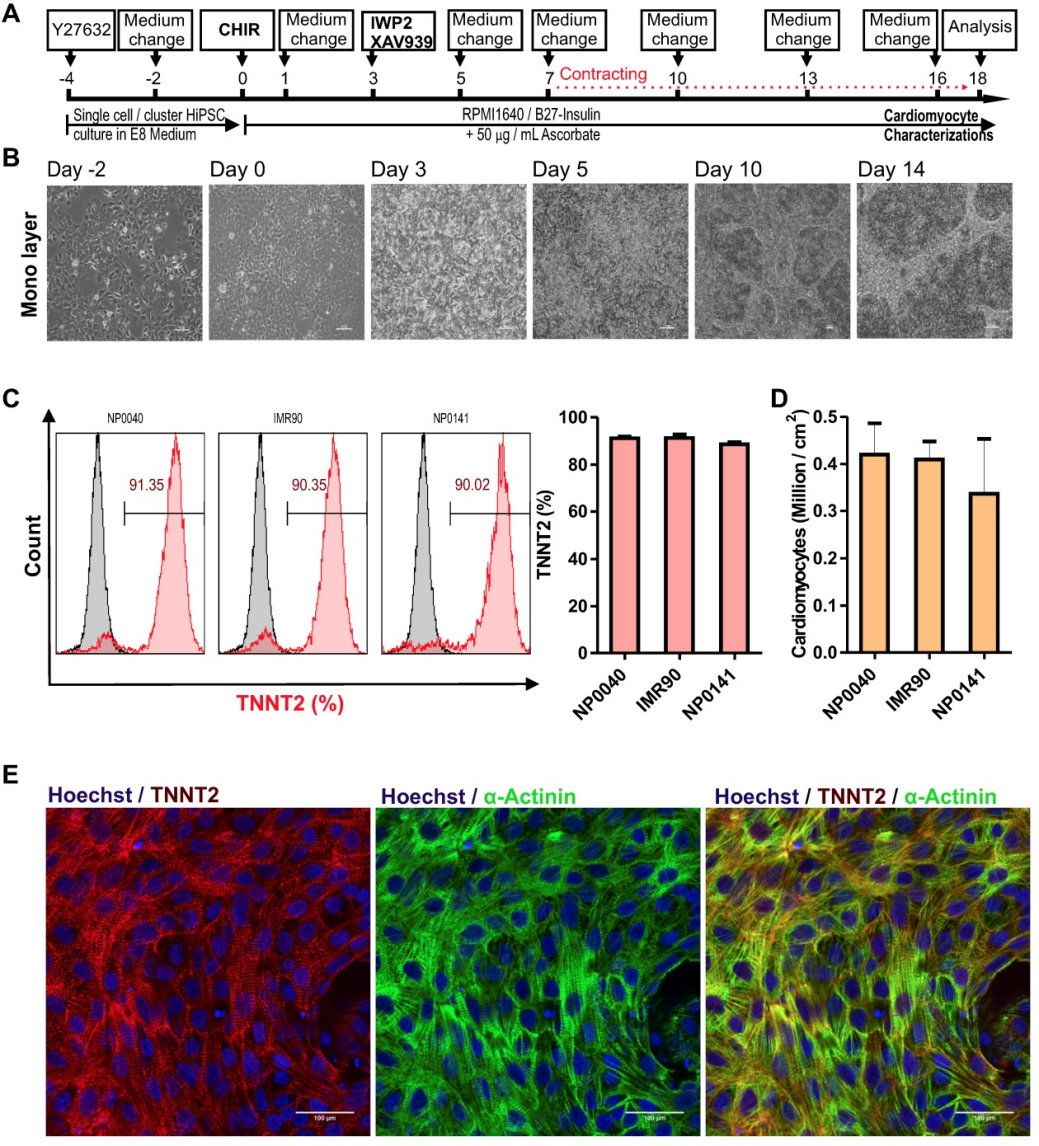
** Cardiac differentiation of hiPSCs in a monolayer culture.** (A) Workflow of cardiac differentiation of hiPSCs. The numbers above the line represent the differentiation days. hiPSCs were cultured in E8 medium from day -4 to day 0 and in RPMI 1640 medium supplemented with 1× B27 without insulin and 50 μg/mL ascorbate from day 0 to day 18. Culture medium was supplemented with 5 μM ROCK inhibitor from day -4 to day -2, with 8 μM CHIR99021 from day 0 to day 1, and with 5 μM IWP2 and 5 μM XAV939 from day 3 to day 5. (B) Brightfield images of monolayer cultures of differentiating cells at 10X magnification. Scale bars: 100 μm. (C) Left histogram charts show representative flow cytometric analysis of hiPSC-CMs at day 18 of differentiation. Grey histograms represent isotype controls and the red histogram samples stained with TNNT2 antibodies and numbers inside the graphs represent the percentage of TNNT2-positive cells, and right bar chart is flow cytometry data analysis of biological independent replications for NP0040 (n = 6), IMR90 (n = 4) and NP0141 (n = 3) hiPSC-CMs at day 18 of differentiation. (D) Cardiomyocyte yields per cm^2^ growth area in NP0040, IMR90, and NP0141 hiPSC lines. Data are shown as mean ± SD (n = 6, 4, and 3, respectively). (E) Immunocytochemical analysis of NP0040 hiPSC-CMs that were differentiated on Matrigel-coated cover glass. Cells in monolayer cultures were stained without prior dissociation with antibodies against TNNT2 (red) and α-actinin (green). Nuclei were counterstained with Hoechst 33342 (blue). Scale bars: 100 μm.

Monolayer cultures are easily accessible to most laboratories that are not equipped with suspension bioreactor technologies. Therefore, our priority aim was to standardize hiPSC-CMs differentiation under monolayer conditions to achieve high reproducibility, differentiation efficiency and CM yield. Following the protocol depicted in **Figure 1A**, hiPSC cell lines were cultured in E8 medium for four days prior to induction of cardiac differentiation in order to generate a confluent cell layer **(Figure 1B)**.

The treatment of three different hiPSC lines (NP0040, IMR90, and NP0141) for the initial 24 h with CHIR99021 at the concentration of 6 to 8 μM followed by treatment with IWP2 and XAV939 from days 3 to 5 was sufficient to reach a maximal cardiomyocyte induction with >90% TNNT2-positive hiPSC-CMs on day 18 of differentiation (**Figure 1C**). CHIR99021 concentration range was chosen based on a titration experiment with increasing concentrations of CHIR99021 indicating that increasing CHIR99021 concentration to 10 and 12 μM did not further enhance differentiation. However, the efficiency of hiPSC-CM differentiation was reduced to 50.9 ± 17.0% and 61.9 ± 24.2% (n = 4) at CHIR99021 concentrations of 2 and 4 μM, respectively (**Figure S1**).

Cardiomyocyte contractions were first observed by light microscopy at day 7 of differentiation **(Movies S1a** and** S1b)** and were easily observed by naked eye without microscopic magnification from day 8 onwards **(Movie S2)**. This protocol is referred to as standard protocol in the following sections.

Flow cytometric analysis revealed that cardiac differentiation of NP0040, IMR90, and NP0141 iPSC lines in monolayer cultures yielded cell preparations containing 91.2 ± 0.7% (n = 5), 91.3 ± 1.6% (n = 4), and 88.6 ± 1.6% (n = 3) TNNT2-positive CMs, respectively (**Figure 1C, right panel**). The total cardiomyocyte yield in these differentiations was 0.42 ± 0.07 x 10^6^ per cm^2^ growth area (n=5) for NP0040, 0.41 ± 0.04 x 10^6^ per cm^2^ growth area (n=4) for IMR90, and 0.34 ± 0.12 x 10^6^ per cm^2^ growth area (n=3) for NP0141 iPSC lines **(Figure 1D)**.

In order to assess the structural organization of sarcomeric proteins in CMs, 0.6 × 10^6^ NP0040 undifferentiated iPSCs were seeded on glass cover slips and the standard protocol was applied for cardiomyocyte differentiation. On day 18, double immunostaining was performed for TNNT2 and α-actinin, which showed that these cardiac proteins are expressed in CMs with a high degree of sarcomeric organization **(Figure 1E)**.

### Simultaneous inhibition of Wnt signaling by ascorbate, IWP2, and XAV939 increases the reproducibility and efficiency of hiPSC-CM differentiation

**Figure 2:**
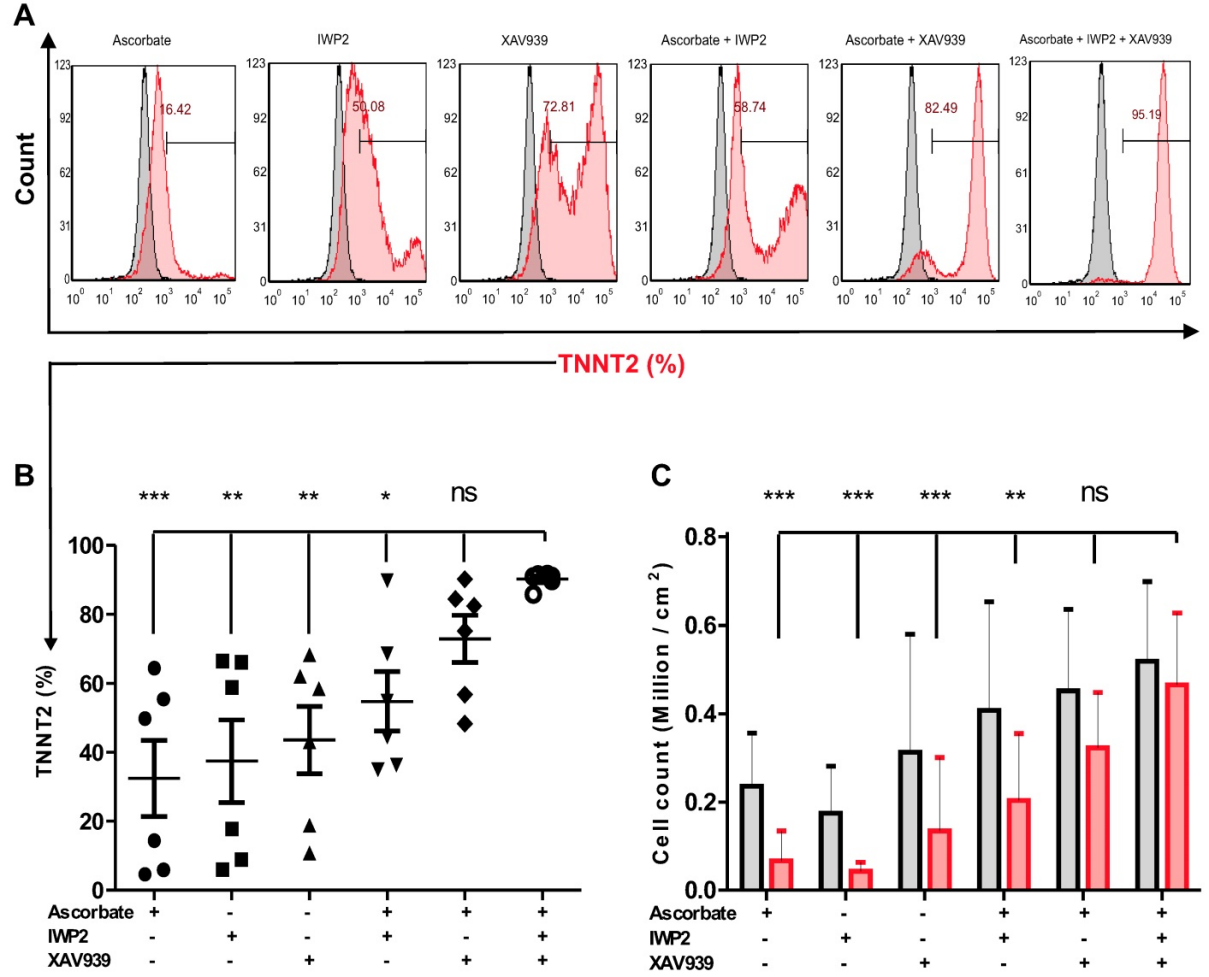
** The effect of different combinations of small molecules on cardiac differentiation efficiency of hiPSCs.** (A) NP0040 hiPSCs were seeded at the initial density 0.6 cells/cm^2^ and differentiated in a monolayer culture in basal differentiation medium consisting of RMPI 1640 and 1× B27 supplement. In all experimental groups, the cardiac differentiation was induced at day 0 by addition of 8 μM CHIR99021 for 24 h. In all ascorbate containing groups, the ascorbate was present in the medium from day 0 until the end of differentiation. Cells were treated with IWP2 and XAV939 alone or in combination from day 3 to day 5 of differentiation. Flow cytometry analysis was performed at day 18 of differentiation. (A) Cells were stained with isotype (grey histograms) or TNNT2-specific antibodies (red histogram) and the percentage of TNNT2-positive cells in each experimental group is indicated in the panels. (B) Percentages of TNNT2-positive cells in different experimental groups are shown in panel B. (C) Total live cell number (black) and cardiomyocyte number (red) per cm^2^ growth area. Data in B and C are shown as mean ± SD of six independent biological replicates. Statistics were performed by one-way ANOVA with Bonferroni's multiple comparison post hoc. P < 0.05 was considered as a statistically significant difference.

To test the efficiency and reproducibility of the cardiomyocyte differentiation protocol, biologically independent experiments (n = 6) with various passage numbers of NP0040 hiPSCs, and different combinations of small molecules IWP2 and XAV939 and ascorbate were compared. **Figure 2A** shows representative flow cytometric data. These analyses showed that the continuous supplementation of ascorbate to the basal differentiation medium (RPMI 1640 + 1× B27) and addition of 8 μM CHIR99021 for the initial 24 h, but lack of treatment with IWP2 and XAV939 during days 3-5 of differentiation, poorly induced cardiomyocyte differentiation (32.5±27.1%, n=6) (**Figure 2A,B)**. In contrast, the omission of ascorbate and the addition of either IWP2 or XAV939 to the basal medium during days 3-5 of differentiation increased the percentage of TNNT2-positive cells at day 18 of differentiation to 37.4 ± 29.4% and 43.6 ± 24.0% (n = 6), respectively (**Figure 2A,B**). The most efficient and robust differentiation was achieved by combining IWP2 and XAV939 for 48 hours from day 3 to day 5 with continuous ascorbate supplementation throughout the whole differentiation process **(Figure 2A,B)**. All three factors synergistically increased the efficiency and reproducibility of cardiomyocyte differentiation to 90.2 ± 2.2% (n = 6) **(Figure 2A,B)**. The total cardiomyocyte yield was 0.51 ± 0.14 ×10^6^ CMs per cm^2^ growth area when ascorbate, IWP2 and XAV939 were combined (n=6) (**Figure 2C)**.

### Effect of seeding density on the efficiency of hiPSC-CM differentiation

Previous protocols show variations in the differentiation efficiency when suboptimal densities of PSCs are used in the initial stages of the differentiation protocol 21, 22, 24. To compare the state-of-the-art with our approach we adapted a previously reported differentiation protocol to generate hiPSC-CMs 22, and found that this protocol is sensitive for different initial seeding densities at the start of differentiation: Nine seeding densities including 0.01, 0.02, 0.04, 0.06, 0.08, 0.1, 0.2, 0.3 and 0.4 × 10^6^ cells per cm^2^ of NP0040 cell line were used and induced to differentiate to hiPSC-CMs. At day 20 the fraction of TNNT2 positive cells was analyzed by flow cytometry in four biological independent replications. Representative data from these analyses are shown in **Figure S2A**. Quantification of CM-purity indicates that 59.5 ± 9.6% (n = 4) and 56.4 ± 14.1% (n = 4) of TNNT2-positive cells were obtained at iPSC seeding densities of 0.3 and 0.4x10^6^ cells per cm^2^, respectively. These values differed significantly (P < 0.05) from TNNT2 positive cells obtained in differentiation experiments starting with a seeding density of 0.01x10^6^ cells per cm^2^ (26.4 ± 16.0%, n = 4) (**Figure S2A**. There was no statistical difference in cardiomyocyte yield between nine seeding densities (**Figure S2B**). In addition the maximally achievable CM yield per cm^2^ of culture substrate (“yield max”) was approximated by nonlinear regression: 0.1 ± 0.06x10^6^ CMs/cm^2^ (n = 4) was yield max for seeding density of 0.02 x 10^6^ hiPSCs / cm^2^ (**Figure S2C**).

**Figure 3:**
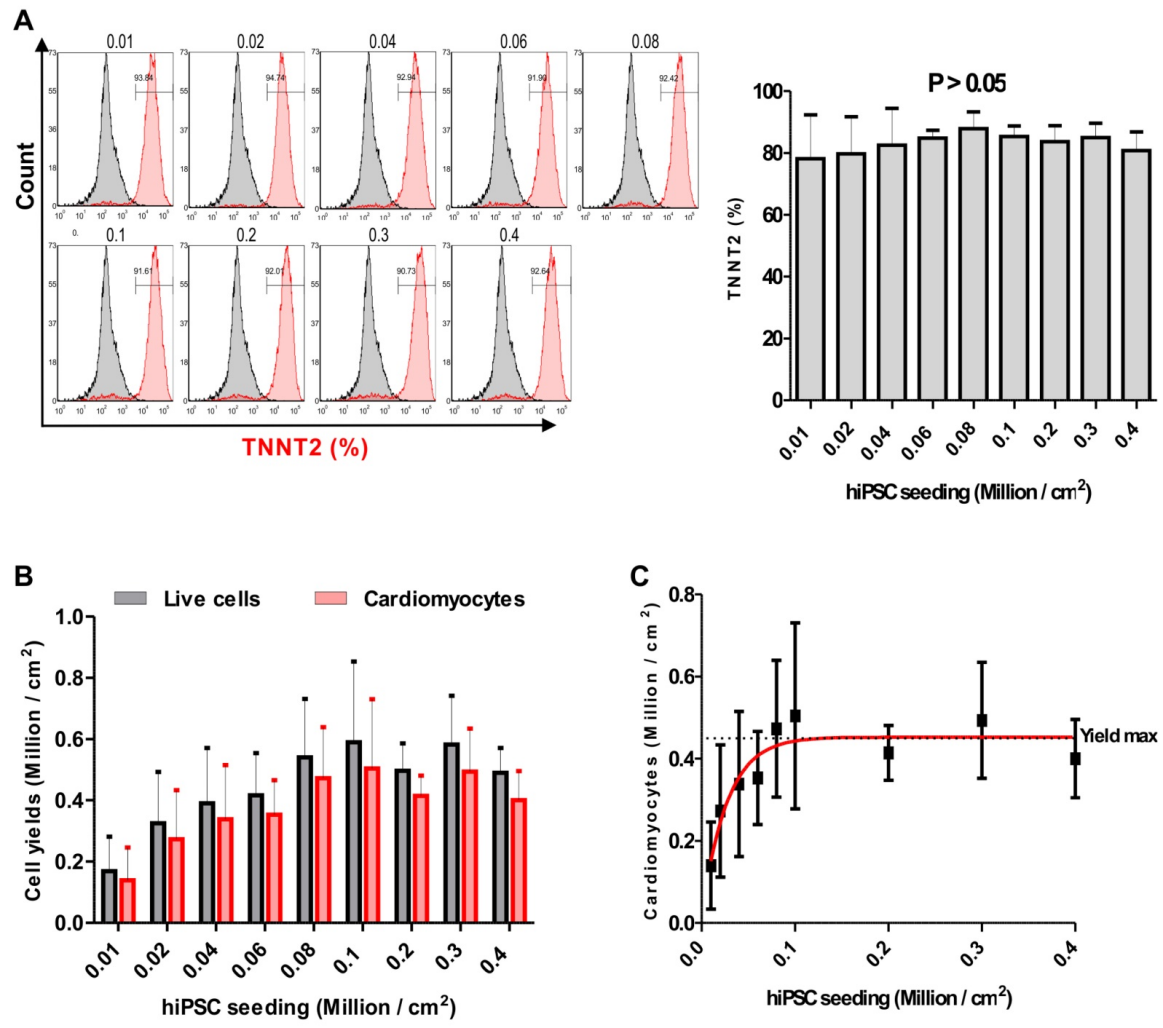
** Effect of the initial hiPSC seeding density on cardiac differentiation.** (A) Flow cytometric analysis was carried out at day 18 of differentiation. Left panel histograms show cells in each group stained with isotype (grey histogram) or TNNT2-specific (red histogram) antibodies. Numbers inside the graphs represent the percentage of TNNT2-positive cells (individual, representative measurements), and right panel bar chart shows statistical analysis of the differences in the percentage of TNNT2-positive cells in nine seeding density groups. Data are shown as mean ± SD of five independent biological replicates. Statistical analyses of data were performed by one-way ANOVA with Bonferroni's multiple comparison post hoc. P < 0.05 was considered as a statistically significant difference. (B) Total live cell number (black bars), and cardiomyocyte yield (red bars) per cm^2^ growth area are shown as mean ± SD of five independent biological replicates. (C) Fitting of average cardiomyocyte yields obtained at different seeding densities to a sigmoidal curve. Data are presented as mean ± SD of five independent biological replicates. Yield max indicates maximal cardiomyocyte yield.

In order to determine the effect of the hiPSC seeding density on cardiac differentiation efficiency in our optimized protocol, NP0040 hiPSCs were seeded at day -4 of differentiation at densities of 0.01, 0.02, 0.04, 0.06, 0.08, 0.1, 0.2, 0.3 and 0.4 × 10^6^ cells per cm^2^, and again the CM purity and the yield were determined by flow cytometry in all nine groups. Flow cytometric analysis performed at day 18 of differentiation revealed that the purity and yield of CMs produced by our protocol are less dependent on the initial iPSC seeding density. The CM purity assessed as the fraction of TNNT2-positive cells ranged from 78.1 ± 14.3 % (n = 5) in the 0.01×10^6^ cells/cm^2^ group to 87.8 ± 5.5 % (n = 5) in 0.08×10^6^ cells/cm^2^ group **(Figure 3A left and right panels)**. There was no statistical difference (P > 0.05) in the fraction of TNNT2-positive cells between all nine seeding densities (n=5) **(Figure 3A right panel)**. There was a trend pointing to a continuous decrease in yield of CMs when less than 0.08×10^6^ cells (n = 5) were initially plated per cm^2^ (**Figure 3B**). In order to determine the most efficient conditions, a sigmoidal curve was fitted based on the average cardiomyocyte yield to determine the yield max in this experiment (n = 5). This approximation revealed a maximal yield of 0.48 ± 0.17 (n = 5) ×10^6^ CMs per cm^2^ growth area for all initial cell seeding densities in the range of 0.08 - 0.4×10^6^ cells/cm^2^, and indicated that increasing the starting cell number beyond a certain value does not result in higher cardiomyocyte yields **(Figure 3C).**

### Generation of hiPSC-CMs in 3D bioreactor suspension culture

**Figure 4:**
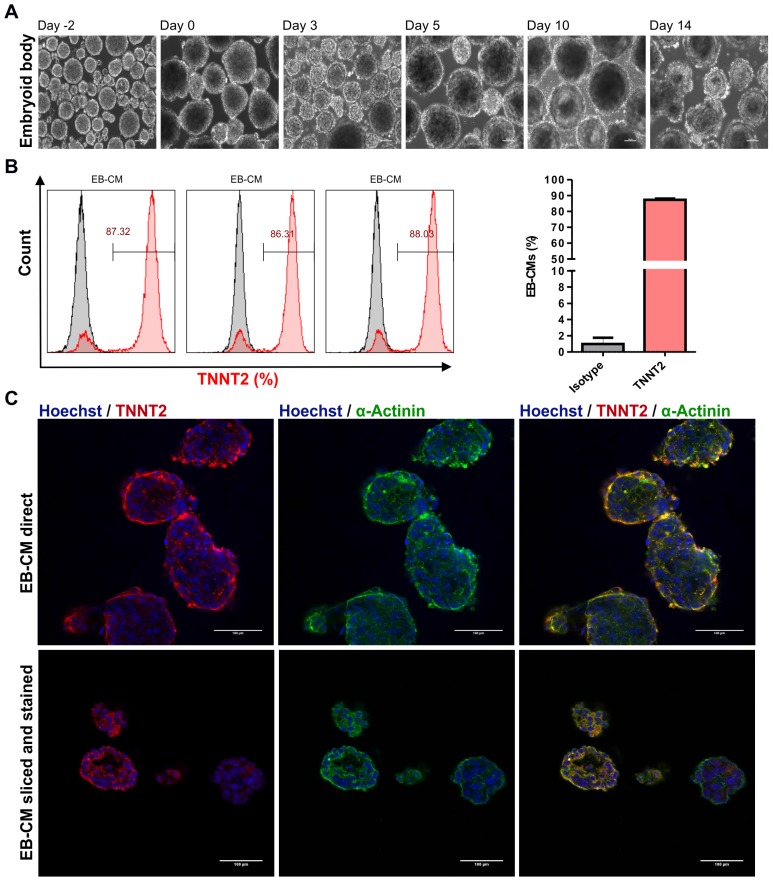
** Differentiation of hiPSCs to cardiomyocytes in bioreactor suspension culture using the optimized protocol.** (A) Bright field images of cell aggregates formed in a suspension culture of NP0040 hiPSCs from day -4 to day 14 at 10X magnification. Scale bars: 100 μm. (B) Left panel histograms show the fraction of TNNT2-positive cells at day 18 (red), analyzed by flow cytometry in 3 different differentiation experiments. Results of isotype controls are shown in grey. Bar chart represents the fraction of positive cells as mean ± SD of three independent biological replicates (C) Expression of TNNT2 (red), and α-actinin (green) in cardiac clusters at day 18 of differentiation. Nuclei were stained with Hoechst 33342 (blue). Images were obtained with a SP8 Leica confocal microscope. Cardiac cluster were stained as whole mounts (upper images), or dissected into 8 µm slices prior to staining (lower images). Scale bars: 100 μm.

To generate large numbers of hiPSC-CMs for preclinical experiments in cardiac regenerative medicine and other applications, scalable technologies need to be applied. To this end, a stirred suspension bioreactor was inoculated with hiPSCs and cells were cultured for four days prior to initiation of differentiation (**Table S1**). In this period, 5 μM of ROCK inhibitor was present in the medium for the first two days to increase the cell survival and support the formation of EBs **(Figure 4A)**. Employing the standard protocol as optimized for 2D monolayer differentiation cultures to the 3D bioreactor culture without further modification resulted in formation of EBs that exhibited spontaneously contracting areas already at day 7 of differentiation **(Movie S3** and** S4)**. To determine cardiomyocyte purity, flow cytometric analysis was performed, and analyses demonstrated that dissociated EBs were composed of 87.2 ± 0.9 % (n = 3) TNNT2-positive CMs at day 18 when an initial inoculum of 40 x 10^6^ hiPSCs was used **(Figure 4B** and** Table 1)**. Additionally, immunostaining confirmed that cardiac bodies contained large fractions of cells that stained positive for the cardiac TNNT2 and α-actinin **(**whole mounts are shown in** Figure 4C upper panel**, and cryosections in** Figure 4C, lower panel)**.

Cardiac bodies from suspension bioreactors were dissociated into single cells, and single cells were cultured on Matrigel-coated 48 wells plates to form monolayers of hiPSC-CMs. After two days of culturing the mono layer hiPSC-CMs were contracting macroscopically similar to hiPSC-CMs generated in the 2D system (**Movie S5**). In addition, cardiomyocyte-yield determined as the total number of TNNT2-positive cells in three independent differentiations, was 71.9 ± 29.8 ×10^6^ CMs per 100 mL bioreactor culture volume for NP0040 hiPSC line **(Table 1).**

### Expression of myosin light chain 2 isoforms

We performed a 60 days follow up experiment with probe sampling every ten days to gain knowledge on the expression kinetics of subtype specific myosin light chain 2 isoforms. Cardiac proteins TNNT2, α-ACTININ, MLC-2v, and MLC-2a were detected by flow cytometry. Flow cytometric analysis indicated that pan-cardiomyocyte markers TNNT2 and α-ACTININ were expressed at low levels on day 10 (30.8 ± 7.7, n = 3), and highly expressed on day 20 (83.6 ± 1.8, n = 3) which remained at the similar level until day 60 (**Figure S3A**). Ventricular myosin light chain isoform MLC-2v was found to be highly expressed on day 10 (84.1 ± 5.2, n = 3), and less expressed on day 20 (65.5 ± 15.1, n = 3) and onwards (**Figure S3B**). Expression of the atrial isoform MLC-2a continuously increased until day 40 (45.0 ± 15.4, n = 3), and decreased on days 50 (24.5 ± 7.1, n = 3) and 60 (23.7 ± 9.0, n = 3) (**Figure S3B**).

### Transcription profiling of hiPSC-CMs

To further characterize the hiPSC-CMs we analysed the expression level of heart specific genes using genome wide DNA microarrays on day 20 of differentiation. It is expected that several heart specific genes such as genes essential for heart contraction would indicate high expression level. Table S2 indicates the expression level (log2 and absolute values) of all human coding genes. Among the 21450 coding genes the expression level varied between the maximal value of 863694±15768 (mean±SD) and the minimal value of 6±1 which corresponds to *ND5* and *LIPK,* respectively. It should be considered that expression values below 64 are considered as “noisy” values. In this context, it should be expected that cardiac specific genes showed high expression levels. Therefore we select the first 100 genes with the highest expression levels (Table S3) and analysed them using the DAVID bioinformatics tools (https://david.ncifcrf.gov/) as described previously 25, 26. As indicated in Table S3, the 100 genes with the highest expression level belong to heart specific Gene Ontologies (GOs). In particular, several cellular component (CC) - and biological processes (BPs) sub-ontologies as well as KEGG pathways which are associated with the heart contraction are statistically highly available. Also, GO BPs (e.g., GO:0042773~ATP synthesis coupled electron transport) and KEGG pathways (e.g., hsa00190:Oxidative phosphorylation) which are specific for the CMs energy metabolism 27 have been identified. As indicated in the Table S3 out of the 100 genes with the highest expression levels several genes are associated with the protein synthesis in ribosomes (e.g., GO:0006414~translational elongation; GO:0005840~ribosome). In this context it was shown that protein synthesis and ribosomal genes are highly expressed in CMs 28, 29. These results demonstrate that heart specific GOs and KEGG pathways are highly enriched into hiPSC-CMs.

### Functional analysis of hiPSC-CMs: Calcium transients

**Figure 5:**
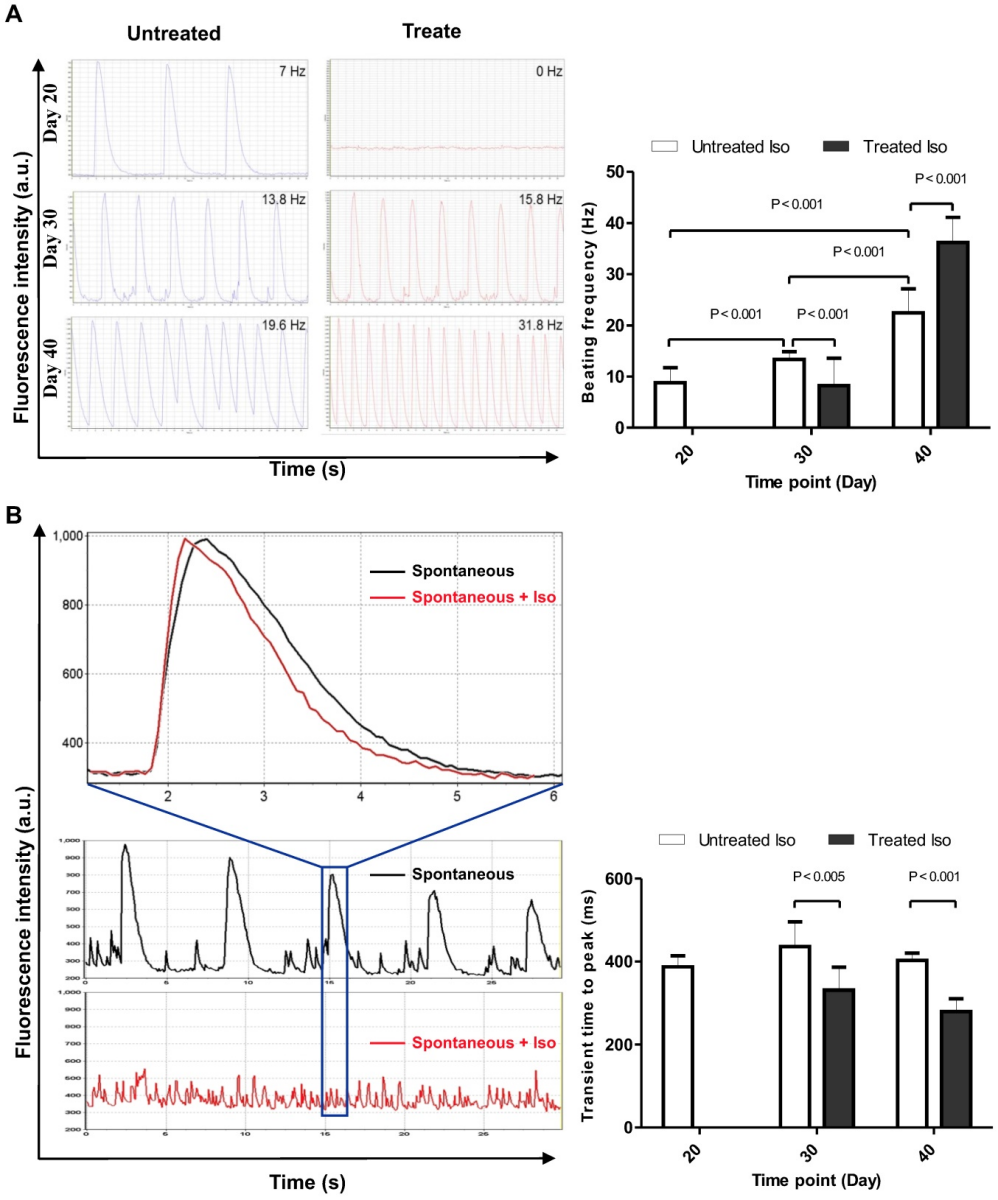
** Calcium imaging of control and 10 µM isoproterenol-treated hiPSC-CMs.** (A) Beating frequency of hiPSC-CM, left panel shows representative data, and right panel displays statistical analysis of ten measurements of spontaneous contraction frequency of hiPSC-CMs. (B) Calcium transient time to peak on day 20, 30, and 40 time points of the differentiation process, left panel shows representative data, and right panel displays statistical analysis of calcium transients in ten hiPSC-CMs. Statistical analyses of data were performed by Student unpaired t test. P < 0.05 was considered as a statistically significant difference.

Calcium ions play important roles in cellular physiology and its contribution in cardiomyocyte physiology can be utilized as a parameter to determine levels of cardiomyocyte maturation at different time points of the differentiation process. Therefore, we performed confocal calcium imaging of hiPSC-CMs on day 20, 30, and 40 of differentiation. The effect of prolonged culture of hiPSC-CMs on frequency and kinetics of contraction-related, global Ca^2+^ transients was examined in the absence (**Video S6**) and presence of adrenergic stimulation by 10µM Iso (**Video S7**). In day 20 hiPSC-CMs, 10 µM Iso abolished spontaneous contractions, which coincided with the dispersion of spontaneous, global Ca^2+^ transients into high frequency local Ca^2+^ release events (**Video S7**), accompanied by an increase in cytosolic resting [Ca^2+^] (**Figure 5a**). In contrast, application of Iso at day 30 and 40 did not interfere with the presence of global Ca^2+^ transients but led to an increase in beating frequency. A statistically significant difference (P < 0.001) was found for the increase in spontaneous beating frequency (beats per minute) between day 20 (8.7 ± 2.9, n = 10), 30 (13.3 ± 1.5, n = 10) and 40 (22.4 ± 4.7, n = 10), as well as between untreated and Iso-treated samples at day 30 (13.3 ± 1.5, n = 10 and 8.2 ± 5.3, n = 10) and day 40 (22.4 ± 4.7, n = 10 and 36.2 ± 4.8, n = 10). To check whether the kinetics of Ca^2+^ release and the responsiveness towards adrenergic stimulation increased with maturation, the calcium transients of hiPSC-CM recoded in the absence and presence of 10 µM Iso were analyzed for the *time-to-peak* (ttp) parameter (**Figure 5b**). In both, day 30 and day 40 cells, Iso led to an acceleration of Ca^2+^ release kinetics: ttp = 436±60 ms (n=9) vs 332±54 ms (n=7) at day 30 and ttp = 403±17 ms (n=10) vs 280±31 ms (n=10) at day 40.

### Contraction frequency and hormonal response of 2D vs. 3D differentiated hiPSC-CMs

**Figure 6:**
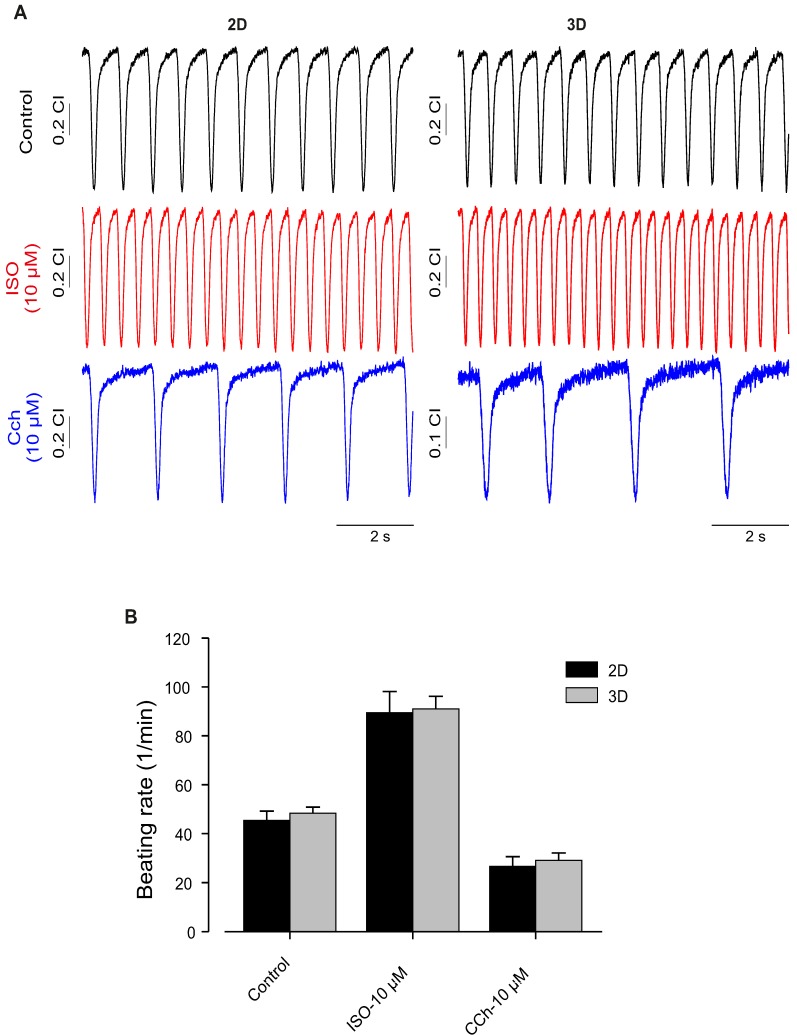
** Functional characterization of hiPSC-CMs cultured in 2D and 3D conditions.** (A) Typical examples of spontaneously beating signal demonstrating similar positive chronotropy after administration of the β-adrenergic agonist Iso (10 μmol/L) in both culture conditions. Likewise, application of carbachol (10 μmol/L) reduced rhythmic beating activity of the CMs, demonstrating functional expression and integration of β-adrenergic and muscarinic signaling in both 2D and 3D conditions. (B) Bar graph shows no differences between CMs of 2D and 3D. Beating rate was determined using the xCELLigence RTCA Cardio software version 1.0 at threshold 12. Error bars indicate mean ± SD of at least 8 wells (8 technical replicates) of each experiment (n=2).

Furthermore, as one of the important factors of normal cardiac function is the intact response to different stimulations, we evaluated effects of Iso and muscarinic cholinergic receptor antagonist carbachol (CCh, 10 µM) on beating activity of hiPSC-CMs generated under 2D and 3D. This experiment was conducted to compare the quality of CMs generated by these different approaches. As shown in **Figure 6**, application of Iso induced significant increase of beating activity (**Figure 6A**, middle panel) compared with control beating rate (**Figure 6A**, upper panel) of hiPSC-CMs from 2D and 3D systems. The application of CCh evoked a significant reduction of beating frequency in both 2D and 3D hiPSC-CMs (**Figure 6A**, lower panel). These results revealed no differences between both 2D and 3D differentiation systems and suggest the presence of intact of ß-adrenergic and muscarinic signaling pathways (**Figure 6B**).

### Electrophysiological characterization of differentiated hiPSC-CMs

**Figure 7:**
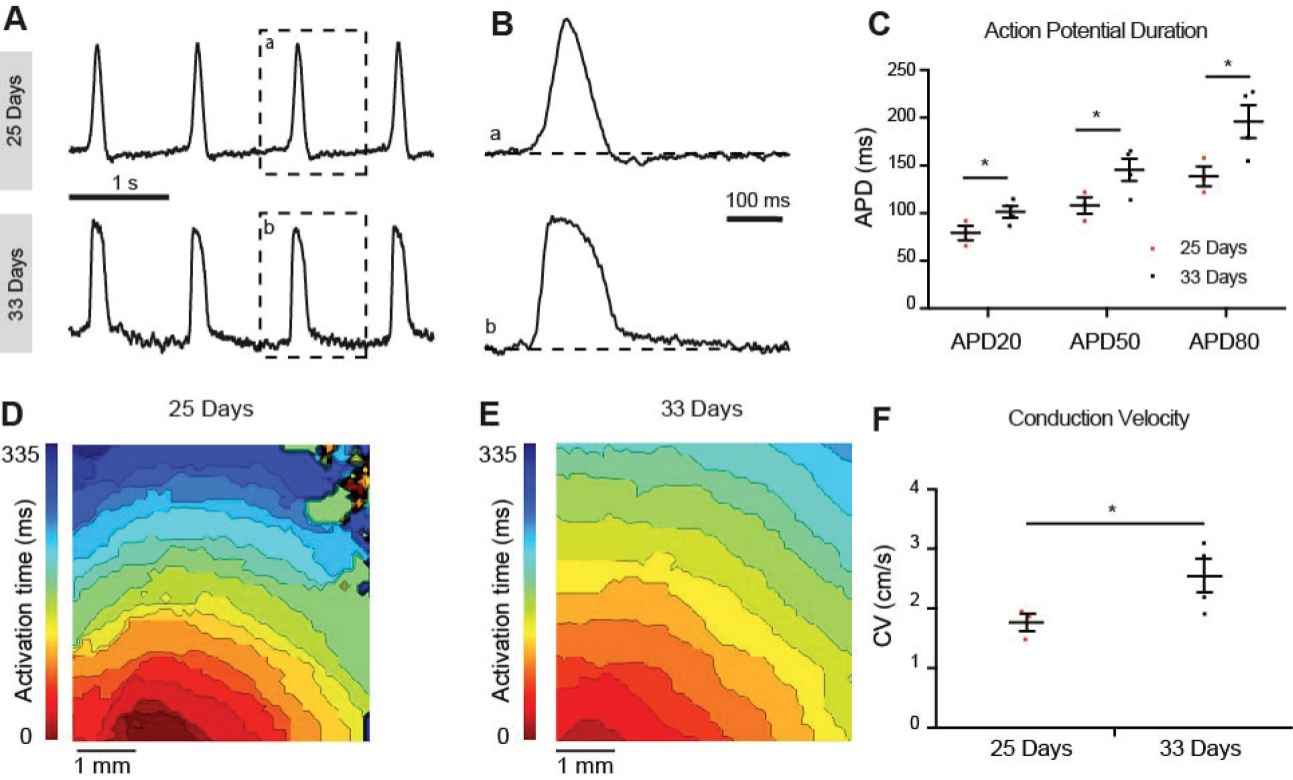
** Optical membrane potential mapping of hiPSC-CMs.** (A,B) Action potentials were measured by voltage sensitive dyes at day 25 and 33 of differentiation and (C) action potential parameters were analyzed. (D,E) Optical mappings of excitation spread in layers of hiPSC-CMs at day 25 and 33 and (F) calculation of conduction velocities.

To determine the maturation status we recorded optical action potentials of monolayers of hiPSC-CMs at 25 and 33 days of differentiation. At 25 days the hiPSC-CMs showed spontaneous phase 4 depolarization but not at 33 days after differentiation (Figure 7A). Spontaneous beating rate was higher at 33 days compared to 25 days but this difference did not reach statistical significance (52±12 vs 38±12 bpm respectively, p=0.21). Action potential duration at 20%, 50% and 80%, however, was longer at 33 days compared to 25 days (Figure 7B). Conduction velocity was faster at 33 days compared to 25 days (Figure 7F). Taken together, the hiPSC-CMs acquired a more ventricular phenotype upon differentiation. Conduction velocity, however, remained slower than in mature ventricular myocardium.

## Discussion

The protocol provided in this study allows for robust and cost-efficient generation of hiPSC-CMs by continuous supplementation of ascorbate in the differentiation medium and combined treatment with IWP2 and XAV939 during day 3 to 5 of differentiation. We showed that this triple combination increases the efficiency and reproducibility of CM differentiation. Furthermore, our data demonstrates that combined inhibition of porcupine and tankyrase sub-pathways of Wnt signaling with IWP2 and XAV939 results in lower sensitivity of the cardiac differentiation efficiency to initial cell seeding density in monolayer differentiation cultures. Starting from hiPSC seeding densities in a broad range, differentiation was initiated after four days of subsequent culture without further controlling the confluency of the hiPSCs. This achievement is the most important innovation in the present protocol, because it substantially simplifies the differentiation process and eliminates the need to carefully control cell confluency. In contrast to common protocols, insulin was not supplemented throughout the whole differentiation process, thereby, in combination with use of homemade E8, reducing the expenses of the differentiation process.

IWP2 attenuates porcupine Wnt signaling 30. Lian and colleagues utilized IWP2 to temporally manipulate Wnt-mediated β-catenin activation in the differentiation process to induce mesodermal commitment resulting in improved hiPSC-CM generation efficiency ranging from 82% up to 95%. Nevertheless, the protocol remained sensitive for starting seeding density and culture confluence diversity 11. To develop a protocol that is based on small molecules and less sensitive for cell seeding density we have applied this protocol to generate hiPSC-CMs and utilized flow cytometry to analyze cardiac differentiation resulting in differentiation efficiency below 60%. We speculate that this low efficiency might be due to one or more difference between the original protocol of Lian and colleagues and the way it was reproduced in our laboratory: (1) utilizing different hiPSC lines, (2) culturing hiPSCs prior to differentiation in homemade E8 medium instead of mTeSR medium, (3) reducing Matrigel concentration to 10 μg/cm^2^ for coating hiPSC culture plastic ware, and (4) using a concentration of 8 µM CHIR99021 without titration.

XAV939 is a potent tankyrase pathway inhibitor that causes cytoplasmic stabilization of the axin protein 31. Previously, XAV939 has been used by Hwang and colleagues for CM induction, resulting in >80% CM purity 18. Our data indicates that Wnt pathway modulation by IWP2 and XAV939 requires ascorbate to push CM differentiation efficiency to above 90%, and overcome variabilities related to initial cell seeding density making it less expensive and more attractive for routine hiPSC-CM derivation in preclinical studies.

**Figure 8:**
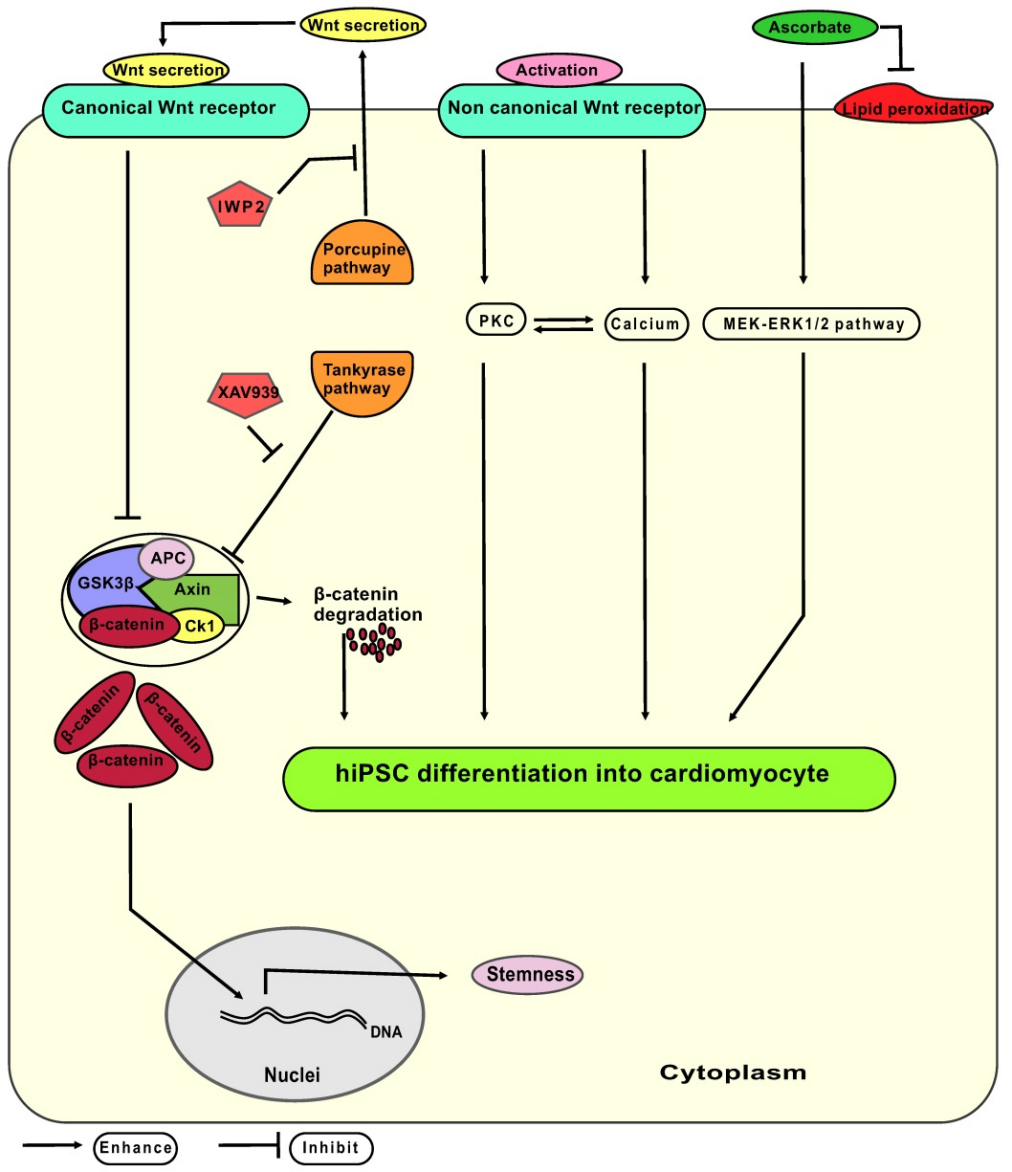
** Hypothetic model of signaling pathways involved in the differentiation of hiPSC-CM from mesodermal progenitors.** Canonical Wnt signaling releases β-catenin from the axin-GSK3β complex, whereas non canonical Wnt signaling acts via activation of the PKC (protein kinase C) pathway and elevates the cytoplasmic calcium levels. The Axin - GSK3β complex consist of the axin protein and GSK3β (glycogen synthesis kinase three β) together with APC (adenomatosis polyposis coli), CK1 (casein kinase 1) and β-catenin. IWP2: small molecule inhibitor of porcupine pathway; XAV939: small molecule inhibitor of tankyrase pathway; MEK-ERK 1/2: mitogen-activated protein kinase-extracellular-signal regulated kinases 1 and 2; ascorbate: L-ascorbic acid phosphate magnesium n-hydrate.

Wnt signaling has a biphasic property on stemness and CM differentiation (hypothetic interplay of signaling pathways: **Figure 8**): On one hand, activation of the Wnt canonical pathway leads to release of β-catenin from axin - glycogen synthase kinase 3 β protein complex, allowing β-catenin to translocate into the nucleus and facilitate maintenance of the stem cells pluripotency network 32, 33. On the other hand, activation of the non-canonical Wnt pathway increases cytoplasmic protein kinase concentrations that enhance cell differentiation 34. Thus, XAV939 and IWP2 synergistically effect on Wnt/β-catenin pathway to enhance CM differentiation and eliminate undifferentiated pluripotent stem cells via accumulating β-catenin inside the cytoplasm. We hypothesized that the non-canonical Wnt pathway remains active during treatment with XAV939 and IWP2 leading to increase in phosphokinase C level, and intracellular calcium concentration, thereby, elevating CM polarization and differentiation.

Combining either IWP2 or XAV939 with continuous ascorbate supplementation increased cardiac fate decision in comparison to groups where no ascorbate was added. Ascorbate seems to enhance the CM differentiation process independently of its antioxidative properties 35. Proposed mechanisms include: (1) Ascorbate induces proliferation of cardiovascular progenitor cells by stimulating MEK-ERK1/2 pathway via increasing collagen synthesis 36-38; (2) Ascorbate induces ten eleven translocation enzyme (Tet) mediated DNA demethylation 39, affecting mesodermal and cardiac progenitor proliferation; (3) Ascorbate enhances differentiation of CMs in early and late stages of cardiomyogenesis by reactive oxygen species, and nitric oxide signaling pathways 40. Potential interactions of ascorbate with Wnt signaling in CM differentiation are not completely understood yet.

Cardiomyocytes can be differentiated from human PSCs, either in monolayer or in suspension cultures 41, 42. Firstly, differentiation in monolayer is routinely used in several laboratories, but can be strongly affected by starting cell seeding density 22, cell line diversity 21, and cell confluency 43. Here we describe for the first time a protocol that lifts obstacles related to initial cell seeding density. This is not only of importance to reduce experimental variability in the production of hiPSC-CMs, but has further implications in cardiac tissue engineering. Based on our protocol new approaches to convert hiPSCs into hiPSC-CMs in more challenging environments such as decellularized cardiac matrix repopulated with hiPSCs could become feasible.

Secondly, scalable suspension bioreactor differentiation is not easy reproducible in each laboratory due to technical challenges such as optimizing agitation speed 44, generating homogenous EB size 24, working load, and substantial costs. Here, we provide a method that is easily transferable from a monolayer system into scalable suspension bioreactor differentiation. We propose to test the protocol with individual hiPSC lines in 2D culture and perform fine-tuning of the process wherever necessary. In the next step the protocol can be adapted to 3D suspension culture without further adaptations.

Despite of overcoming limitations by fluctuations in initial cell seeding density, cell line variations, difficulties in scaling up from 2D to 3D culture systems, reducing high cell culture expenses, and batch to batch variations in hiPSC-CM differentiation, still further investigations are required to find optimal cell dissociation routines and replace Matrigel. Removing Matrigel for coating is mandatory in translational approaches and it is advisable to utilize recombinant laminins instead.

Our data is in line with previously published protocols showing that hiPSC-CMs express several human CM-specific markers including TNNT2, α-actinin, MLC-2v, and MLC-2a, as well as proving physiological calcium transients, and responses to beta adrenergic receptor stimulation 21, 22, 24, 45, 46. In detail, our results indicate that hiPSC-CMs derived from either 2D or 3D differentiations do not differ in their spontaneous beating activity when measured in 2D cell layers and show identical response to adrenergic or muscarinergic receptor stimulation. Analysis of calcium handling in hiPSC-CMs for up to 40 days provided signs of maturation as calcium transients became faster with time and reaction to adrenergic stimulation developed from a negative response in early stages (day 20) to a positive chronotropic response on day 40. Analysis of MLC-2v and MLC-2a may point to a pronounced development of ventricular-like cells from day 40 till day 60.

In summary, temporal manipulation of Wnt signaling via combined inhibition of porcupine and tankyrase pathways with continuously ascorbate supplementation is an effective method for cardiomyocyte differentiation, in both monolayer and suspension culture conditions with less cost, low variabilities, and >90%TNNT2 positive cells. This method should ease the translation of basic research to large animal experiments to pave the road for clinic trials and allow more laboratories to adapt the differentiation technology in reasonable time.

## Supplementary Material

Supplementary Figures, Table S1.

Supplementary Tables S2-S3.

Supplementary Movies.

## Figures and Tables

**Table 1:** Yields and efficiencies of hiPSC-CM differentiation in 3D bioreactor culture with initial inoculation of 40 million hiPSC

Cell line	hiPSC inoculation (Millions / 125 mL E8 medium); day 0	CM yield (Millions / mL RPMI1640 medium); day 18	CM yield (Millions / 100 mL RPMI1640 medium); day 18	TNNT2 positive cells (%)
NP0040	40	0.92	91.7	87.32
	40	0.87	86.4	86.31
	40	0.38	37.6	88.03
**Mean ± S.D**	40	0.73 ± 0.3	71.90 ± 29.82	87.22 ± 0.87

Scalable suspension culture of human induced pluripotent stem cell of NP0040 cell lines differentiated into cardiomyocyte. Abbreviations TNNT2 is of cardiac troponin T, hiPSC is of induced pluripotent stem cells, and CM is of cardiomyocyte. n = 3 biological independent replications.
