# Supplementary material for: Generation of human induced pluripotent stem cell-derived cardiomyocytes in 2D monolayer and scalable 3D suspension bioreactor cultures with reduced batch-to-batch variations
Source: Theranostics. 2019 Sep 25;9(24):7222–38. doi: 10.7150/thno.32058 (PMC6831300; doi:10.7150/thno.32058)
Supplement: Supplementary file 1 — Supplementary Figures, Table S1. [file thnov09p7222s1.pdf]

Suppl. Figure 1: Optimization of CHIR99021 concentration. Flow cytometric analysis was carried out at day 18 of differentiation. Left histograms shows cells in each group were stained with isotype (grey histogram) or TNNT2-specific (red histogram) antibodies and the numbers inside the graphs represent the percentage of TNNT2-positive cells, and right bar chart shows statistical analysis of the differences in the percentage of TNNT2-positive cells in six concentration of CHIR99021 in micro molarity. Data are shown as mean ± SD of four independent biological replicates. Statistical analyses of data were performed by one-way ANOVA with Bonferroni's multiple comparison post hoc. $P < 0.05$ was considered as a statistically significant difference.

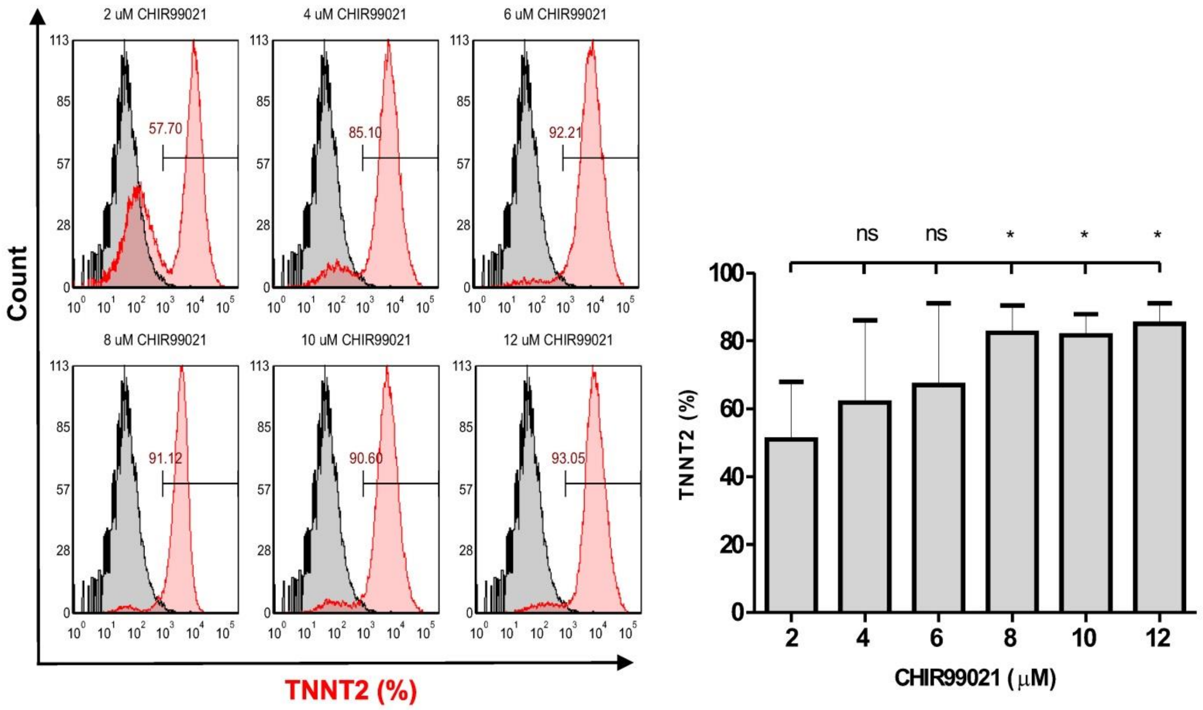

Suppl. Figure S2: Effect of the initial hiPSC seeding density on cardiac differentiation by applying a published protocol. (A) Flow cytometric analysis was carried out at day 18 of differentiation. Left histograms shows cells in each group were stained with isotype (grey histogram) or TNNT2-specific (red histogram) antibodies and the numbers inside the graphs represent the percentage of TNNT2-positive cells, and right bar chart shows statistical analysis of the differences in the percentage of TNNT2-positive cells in nine seeding density groups. Data are shown as mean ± SD of four independent biological replicates. Statistical analyses of data in panels A right bar chart and B were performed by one-way ANOVA with Bonferroni's multiple comparison post hoc. $P < 0.05$ was considered as a statistically significant difference. (B) Total live cell number (black bars), and cardiomyocyte yield (red bars) per cm^2 growth area are shown as mean ± SD of five independent biological replicates. (C) Fitting of average cardiomyocyte yields obtained at different seeding densities to a sigmoidal curve. Data are presented as mean ± SD of four independent biological replicates. Yield max indicates maximal cardiomyocyte yield.

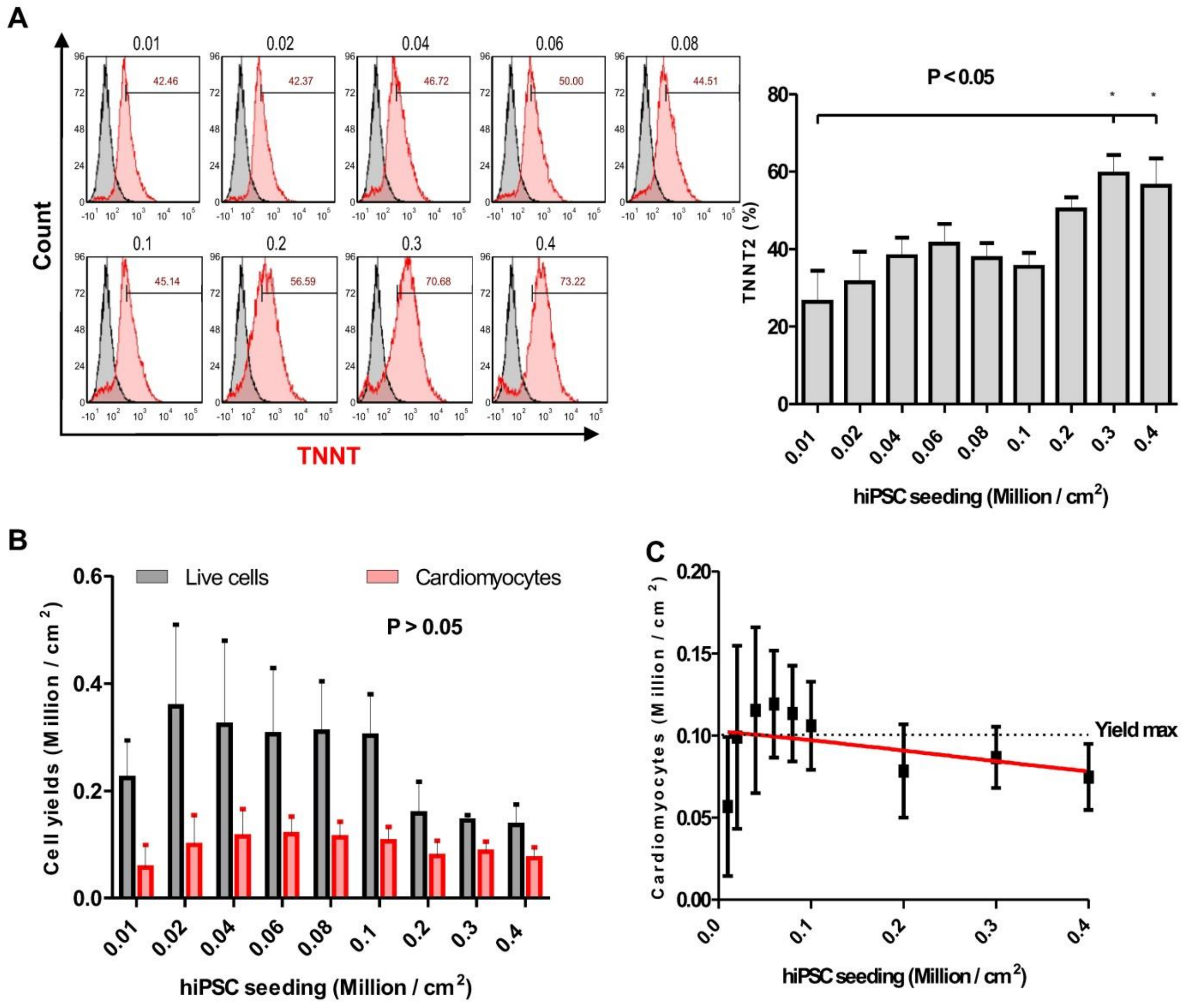

Suppl. Figure S3: Determination and following up of hiPSC-CM sub-types by immunostaining of cardiac protein marker of six time points of the differentiation process. (A) Flow cytometric analysis was carried out at day 10, 20, 30, 40, 50, and 60 of differentiation time points. Left scatter plot shows cells in each time point were unstained (grey dots) or double stained for TNNT2:α-Actinin (red dots) antibodies and the numbers inside the graphs plot in the upper right square represent the percentage of TNNT2:α-Actinin positive cells inside NP0040-CM, and right bar chart shows mean ± SD of three independent biological replicates in the percentage of TNNT2:α-Actinin-positive cells in six differentiation time points. (B) Flow cytometric analysis was carried out at day 10, 20, 30, 40, 50, and 60 of differentiation time points. Left scatter plot shows cells in each time point were unstained (grey dots) or stained (red dots) for Mlc2v in the lower right square, and Mlc2a in upper right square inside the graphs plot represent the percentage of Mlc2v and Mlc2a positive cells NP0040-CM, respectively and right bar chart shows mean ± SD of three independent biological replicates in the percentage of Mlc2v and Mlc2a-positive cells in six differentiation time points.

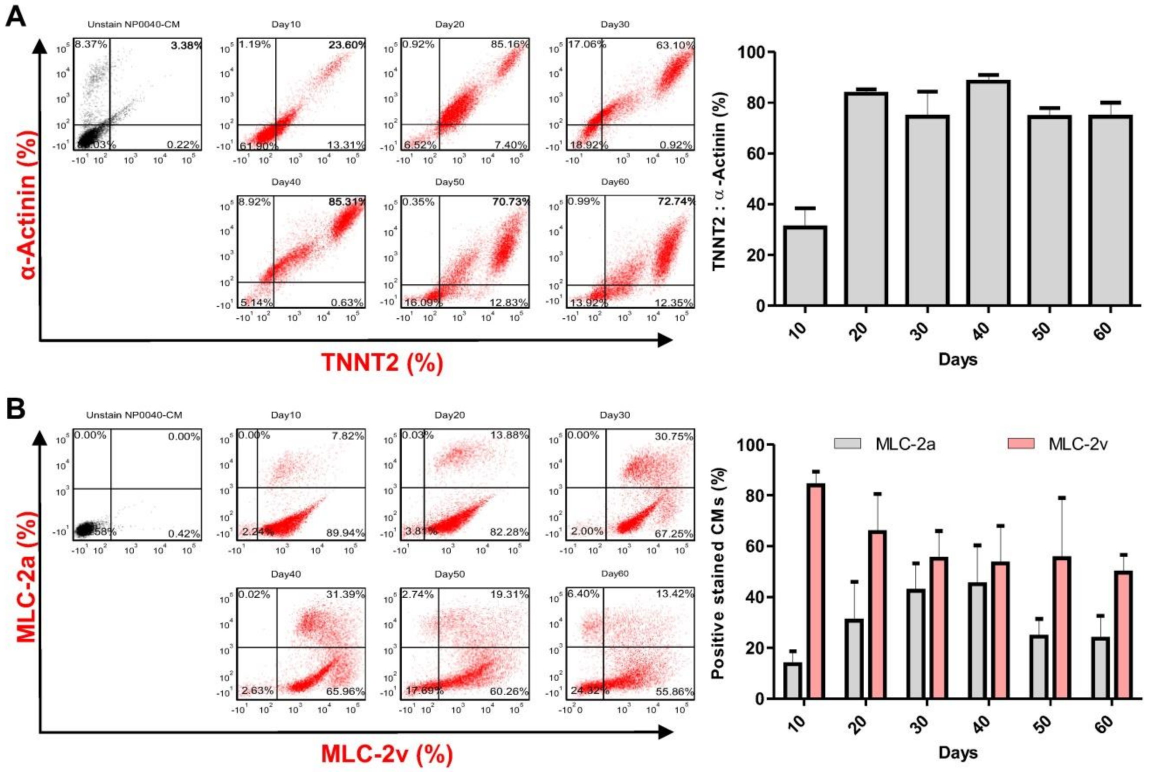

Table S1: Pilot test to determine yields and efficiencies of bioreactor cardiomyocyte differentiation with different hiPSC inoculations

Cell line	hiPSC inoculation (Millions / 125 mL E8 medium); day 0	CM yield (Millions / mL RPMI1640 medium); day 18	CM yield (Millions / 100 RPMI1640 medium); day 18	TNNT2 positive cells (%)
NP0040	37.5	0.475	47.46	79
	**40**	**0.92**	**91.7**	**87.3**
	50	0.494	49.38	83.45
**Mean ± S.D**	42.50 ± 6.614	0.629 ± 0.241	62.84 ± 24.99	83.25 ± 4.154

Scalable suspension culture of human induced pluripotent stem cell of NP0040 cell lines differentiated into cardiomyocyte. Abbreviations TNNT2 is of cardiac troponin T, hiPSC is of induced pluripotent stem cells, and CM is of cardiomyocyte.

Movie S1: (a) and (b): Light microscope movie records of contracting mono layer cardiomyocytes at 100 µm scale bar.

Movie S2: Without light microscope movie records of contracting cardiomyocytes.

Movie S3: Light microscope movie records of contracting bioreactor embryoid body cardiomyocyte at 100 µm scale bar, and low magnification 4X.

Movie S4: Light microscope video records of contracting bioreactor embryoid body cardiomyocyte at 100 µm scale bar and low magnification 10X

Movie S5: Light microscope movie records of contracting EB-CM mono layer cardiomyocytes after 48 hours of dissociating and platating on the pre coated Matrigel 48 well plates at 100 µm scale bar.

Movie S6: hiPSC-CM calcium imaging of spontaneous contractions.

Movie S7: hiPSC-CM calcium imaging of spontaneous contractions plus 10 µM isoproterenol treatment.
